# Impact of nanopore confinement on phase behavior and enriched gas minimum miscibility pressure in asphaltenic tight oil reservoirs

**DOI:** 10.1038/s41598-024-64194-2

**Published:** 2024-06-11

**Authors:** Fatemeh Keyvani, Ali Safaei, Yousef Kazemzadeh, Masoud Riazi, Jafar Qajar

**Affiliations:** 1 Department of Petroleum Engineering, School of Chemical and Petroleum Engineering, Shiraz University, Shiraz, 7193616511 Iran; 2 Fouman Faculty of Engineering, College of Engineering, University of Tehran, Tehran, 4358139115 Iran; 3 Enhanced Oil Recovery (EOR) Research Centre, IOR/EOR Research Institute, Shiraz University, Shiraz, 7193616511 Iran; 4 Department of Petroleum Engineering, Faculty of Petroleum, Gas, and Petrochemical Engineering, Persian Gulf University, Bushehr, 7516913817 Iran; 5 School of Mining and Geoscience, Nazarbayev University, Kabanbay Batyr 53, 010000 Astana, Kazakhstan; 6 Department of Earth Sciences, Faculty of Geosciences, Utrecht University, 3584 CS Utrecht, The Netherlands

**Keywords:** Minimum miscibility pressure, Miscible gas injection, Phase behavior, Equation of state, Confinement effect, Asphaltene, Chemical engineering, Crude oil

## Abstract

Miscible gas injection in tight/shale oil reservoirs presents a complex problem due to various factors, including the presence of a large number of nanopores in the rock structure and asphaltene and heavy components in crude oil. This method performs best when the gas injection pressure exceeds the minimum miscibility pressure (MMP). Accordingly, accurate calculation of the MMP is of special importance. A critical issue that needs to be considered is that the phase behavior of the fluid in confined nanopores is substantially different from that of conventional reservoirs. The confinement effect may significantly affect fluid properties, flow, and transport phenomena characteristics in pore space, e.g., considerably changing the critical properties and enhancing fluid adsorption on the pore wall. In this study, we have investigated the MMP between an asphaltenic crude oil and enriched natural gas using Peng-Robinson (PR) and cubic-plus-association (CPA) equations of state (EoSs) by considering the effect of confinement, adsorption, the shift of critical properties, and the presence of asphaltene. According to the best of our knowledge, this is the first time a model has been developed considering all these factors for use in porous media. We used the vanishing interfacial tension (VIT) method and slim tube test data to calculate the MMP and examined the effects of pore radius, type/composition of injected gas, and asphaltene type on the computed MMP. The results showed that the MMP increased with an increasing radius of up to 100 nm and then remained almost constant. This is while the gas enrichment reduced the MMP. Asphaltene presence changed the trend of IFT reduction and delayed the miscibility achievement so that it was about 61% different from the model without the asphaltene precipitation effect. However, the type of asphaltene had little impact on the MMP, and the controlling factor was the amount of asphaltene in the oil. Moreover, although cubic EoSs are particularly popular for their simplicity and accuracy in predicting the behavior of hydrocarbon fluids, the CPA EoS is more accurate for asphaltenic oils, especially when the operating pressure is within the asphaltene precipitation range.

## Introduction

Gas injection has been widely used as an efficient enhanced oil recovery (EOR) method^1,2^. In this method, fluid flow and displacement type need to be considered in addition to the economic evaluations. There are three types of gas injection: miscible, immiscible, and near miscible. Experience has shown that miscible gas injection increases production efficiency. In this case, the minimum miscibility pressure (MMP) between oil and gas is a key parameter^2–4^. Various methods have been used to quantify the MMP, including experimental techniques^5–8^, empirical correlations^9–11^, and numerical simulations^12^. Experimental techniques are usually costly and time-consuming. On the other hand, the proposed empirical correlations can be used in a limited range of operating conditions. Consequently, numerical simulation methods are promising tools that can reliably predict the MMP.

Unconventional resources, such as shale and tight reservoirs, have emerged as critical energy resources and are widely distributed worldwide^13^. Implementing EOR techniques in the production of unconventional reservoirs has garnered significant interest in recent years. This can be attributed to their remarkable capability to enhance oil recovery. Among the various methodologies utilized for EOR in unconventional reservoirs, miscible gas injection has emerged as a frequently employed and extensively documented approach^14^. Therefore, calculating the miscibility condition in these reservoirs is of special importance.

Unconventional reservoirs are characterized by the presence of nanopores (pores ranging from 2 to 50 nm, and a significant number of pores < 2 nm^15^) alongside naturally low permeability and porosity. This unique combination of properties is a defining characteristic of unconventional reservoirs.The nanopores in unconventional reservoirs cause the fluid phase behavior in porous media to deviate from the bulk phase^16,17^. It occurs because the interaction between molecules and walls, which is often overlooked in the bulk phase, can have a considerable impact on phase behavior due to the comparable size of the molecule and the pore space in nanopores^18^.

In recent years, many studies have experimentally or theoretically investigated the phase behavior of fluid in nanopores, and all of them have confirmed that the phase behavior of fluid in nano-scale porous media is different from that in conventional reservoirs and cannot be described by bulk-phase thermodynamics^19–21^. The deviations occur due to capillary forces, leading to pressure difference between the equilibrium phases in porous media and resulting in different phase equilibrium calculations between the bulk phase and confined fluid. This issue is especially important in nanopores with very high capillary pressures^22^. Moreover, electrostatic interactions, van der Waals forces, and fluid–structure variations have consequences such as adsorption of fluid molecules on the pore wall and shift of critical properties^23–25^. Many authors have extensively used an effect known as the "confinement" effect to address the influences of pore size, adsorption, capillary effect, etc., on fluid behavior^26^.

The confinement effect can cause the shrinkage of phase envelope^17^ and shift of cricondentherm^27,28^ and critical point^29^. Saturation pressures (bubble point and dew point) are other parameters that change under the influence of porous media^30,31^. This issue is so important that some researchers have attempted to develop new models^32^, simulators^33^, and flash calculation algorithms^34,35^ or modified the renowned existing equations of state (EoS)^25,36,37^ to consider the confinement effect. The nanopores confinement effect also affects even large-scale reservoir properties, such as fluid distribution^38^, production, and recovery^39,40^. Like the abovementioned factors, MMP also changes under the influence of the nanopore confinement effect.

Asphaltene precipitation represents a significant occurrence in gas injection process. The injection of gas disrupts the phase equilibrium of the reservoir fluid, leading to the separation of certain components like asphaltene from the bulk phase. With its characteristics of surface activity, asphaltene can accumulate on the interface of oil and gas and influence their miscibility behaviors. Therefore, in addition to confinement effects, asphaltene precipitation also affect miscibility.

Some researchers have investigated the effects of confinement on MMP (Group 1 of Table 1), while some others have examined the effect of asphaltene precipitation on the process of achieving miscibility (Group 2 of Table 1). Effective parameters considered in each study are indicated by asterisk in below. Furthermore, recent advancements in research methodologies, exemplified by the utilization of molecular dynamics (MD) simulations, have been adopted by some researchers to explore the miscibility process at the molecular level, focusing exclusively on intermolecular interactions (Group 3 of Table 1). These studies are discussed in detail in the Supplementary Material (Background section).

**Table 1:** Summary of the current state of knowledge on asphaltenic oil–gas MMP in porous media.

	References	Confinement effects			Asphaltene precipitation
		Capillary pressure	Shift of critical properties	Pore wall effects (adsorption, pore radius, molecule-wall interaction, etc.)	
Group 1	Teklu et al.^24,41^	*	*		
	Wang et al.^42^			*	
	Zhang et al.^43,44^	*	*		
	Zhang et al.^45^	*			
	Zhang et al.^46^			*	
	Mohammad et al.^47^		*	*	
	Song et al.^48,49^	*	*	*	
	Sun and Li^3^	*	*	*	
	Sun and Li^50^	*	*		
Group 2	Doryani et al.^51^				*
	Escrochi et al.^52^				*
	Ghorbani et al.^53^				*
	Hassanpour et al.^54^				*
	Kazemzadeh et al.^55,56^				*
	Lu et al.^57^				*
Group 3	Peng et al.^58^	They investigated the MMP between oil and CO_2_ and only considered molecule–molecule interactions and neglected molecule-wall interactions			
	Cui et al.^59^				

As can be seen in previous studies, the impacts of confinement and asphaltene precipitation have not been investigated simultaneously, but they have significant effects on phase behavior and miscibility. Therefore, it is essential to integrate these two effects into a comprehensive model and thoroughly examine both of them concurrently. EoSs serve as the fundamental basis of thermodynamic models and can be used for a wide range of applications, including chemical and petroleum engineering, environmental and other disciplines^60^. A considerable number of researchers have used the PR EoS to model phase behavior in porous media. This equation is a cubic EoS and is used in most industrial simulators due to its simplicity and accuracy in describing the behavior of hydrocarbon fluids. Cubic EoSs consider only the physical forces between the molecules, while more energetic and complex bonds, such as hydrogen bonds, are also found in hydrocarbon systems. It is also impossible to anticipate asphaltene's presence and precipitation using cubic EoSs^61^. However, asphaltene precipitation and its adsorption play significant roles in fluid phase behavior. On the other hand, although many investigations have addressed the effects of capillary pressure, there is a gap in the adsorption effect on reservoir fluid properties and production performance^62^. PR has been successfully used as a cubic part of CPA EoS by Li and Firoozabadi^63,64^. CPA as an association EoS can consider more complex phenomena such as asphaltene precipitation and can be used to fill this gap. According to the best of our knowledge, the effect of asphaltene precipitation and adsorption on MMP in porous media has not yet been investigated. In this study, the proposed Song algorithm^48,49,65^ is used to calculate the MMP in porous media using PR and CPA EoSs. In this way, both the effects of nanopore confinement and asphaltene precipitation are included in the modeling. Then, the effect of pore radius, type of injected gas, and asphaltene adsorption on MMP are investigated, and the results of both equations are compared. To evaluate the performance of the proposed model, results are compared with the MMP measured by the VIT and slim tube technique.

## Experimental section

### Materials

In this study, live oil has been prepared from one of the fields in southwestern Iran using the bottom-hole sampling method (Table 2). The bubble point pressure of this sample was measured as 16.73 MPa, at 372K, using a constant compositional expansion (CCE) test. Furthermore, the oil composition has been obtained by gas chromatography (GC) analysis (Hydrocarbon compositional analyzer, Vinci Technologies, France), which is presented in Table 2. The injected gases in this study were mixtures of dry gas, natural gas liquid (NGL), and liquefied petroleum gas (LPG), which have been collected from production lines of one of the oil fields in southwestern Iran. The dry gas, NGL, and LPG compositions, which were determined using gas chromatography (GC) analysis, are shown in Table 3. It is worth mentioning that the mixing was performed in order to enrich the dry gas and reduce its MMP. The methodology for preparing the injected gas is described in Section "Method of synthesizing injected gases".

**Table 2:** SARA analysis of oil sample.

	Saturate	Aromatics	Resin	Asphaltene
wt%	45.5	43.6	5.7	5.2

**Table 3:** Composition of injected gases.

Components	Dry gas (mole %)	NGL (mole%)	LPG (mole%)
CO_2_	1.59	0.77	0.58
N_2_	9.53	0.83	0.16
C_1_	80.03	0	0
C_2_	1.96	4.16	8.48
C_3_	0.66	55.23	84.72
iC_4_	0.34	27.97	0.10
nC_4_	0.52	2.33	5.96
iC_5_	0.58	3.01	0
nC_5_	0.36	0.31	0
C_6_	1.95	0.26	0
C_7_	1.45	0.08	0
C_8_	0.84	0.01	0
C_9_	0.17	0	0

### Method of synthesizing injected gases

To make an injectable gas with a specific percentage of richness, it is first necessary to calculate the amount of each gas required at a given pressure and temperature. According to the liquid–gas ratio (LGR), it is determined how much dry gas and how much rich gas (NGL or LPG) should be mixed together under standard conditions. It is worth mentioning that it is not possible to make synthetic gas at standard surface conditions due to the limited volume of the lab devices, so the gas-making process must be performed at high pressures. For this purpose, the amount of gas required to mix under a specific pressure and temperature condition must be calculated. First, a certain amount of dry gas at a specific pressure and temperature condition will be used as a base, and then the amount of rich gas that must be added to the dry gas under the same condition will be calculated. For this, the law of non-ideal gases is applied for both the standard conditions and specific pressure and temperature conditions as follows:

1 $$\frac{PV}{{P_{sc} V_{sc} }} = \frac{ZT}{{Z_{sc} T_{sc} }}$$

where *Z* is the compressibility factor and *P*, *V*, and *T* denote pressure, volume, and absolute temperature, respectively. The subscript *sc* refers to the standard condition. In Eq. (1), the unknown is the standard volume of dry gas, which is obtained by solving the equation in terms of the standard volume of dry gas as follows:

2 $$V_{sc} = \frac{{PVT_{sc} }}{{P_{sc} ZT}}$$

In Eq. (2), the isothermal compressibility factor of the gas under standard conditions is always equal to 1. The standard pressure and temperature are taken to be 1 bar (0.1 MPa) 289 K, respectively. The value of gas compressibility factor at a specific pressure and temperature condition is calculated using the PR EoS and the dry gas composition obtained by gas chromatography analysis. By calculating the standard volume of the dry gas using Eq. (2), the denominator of the fraction in Eq. (1) will be obtained. Then, the standard volume of rich gas that must be mixed with the dry gas under standard conditions is calculated as follows:

3 $$V_{sc,rich} = LGR \times V_{sc,drygas}$$

Having obtained the standard volume required for the rich gas, it is necessary to calculate its volume under specific pressure and temperature condition. To this end, the law of non-ideal gases is rewritten as follows:

4 $$\frac{(P + dP)V}{{P_{sc} V_{sc} }} = \frac{ZT}{{Z_{sc} T_{sc} }}$$

In Eq. (4 )the system pressure is considered to be higher than the dry gas pressure. This is because during the gas synthesis stage, after connecting the cylinders containing two dry and rich gases, for transferring rich gases into the dry gas, the rich gas pressure is slightly higher than the dry gas pressure. This value of the pressure difference between the two gases is usually considered to be equal to 0.7 MPa.

To make injectable gas, first, some dry gas is transferred at a certain pressure and the laboratory temperature to the PVT apparatus (FLUIDEVAL, Vinci Technologies, France). After transferring a certain amount of dry gas into the PVT apparatus, gas pressure, laboratory temperature, and the gas compressibility factor are calculated. Then using Eq. (2)the dry gas standard volume is estimated. Then, using Eqs. (3 and 4), the amount of rich gas that must be added to the dry gas is also calculated. It is also necessary to transfer some of the rich gas to another PVT apparatus, and the rich gas pressure is adjusted to 0.7 MPa, higher than the dry gas pressure. Finally, by connecting two PVT apparatuses, the process of transferring rich gas to dry gas is performed. It should be mentioned that the PVT apparatuses can measure the volume transferred from their cylinder to an accuracy of 0.0001 cc, which greatly increases the accuracy of gas synthesizing. After combining the calculated volumes for the two gases, the combined gas is given enough time to mix thoroughly. The experimental methodology, encompassing both the VIT and slim tube techniques, is comprehensively detailed in the Supplementary Material (Experimental techniques section).

## Modeling section

### Peng-Robinson (PR) *EoS*

The following PR EoS, as modified by Song, et al.^65^, was recommended to account for the adsorption impact:

5 $$P = \frac{RT}{{\frac{{\nu_{m} }}{{\left( {1 - \gamma \beta } \right)}} - b}} - \frac{a}{{\frac{{\nu_{m} }}{{\left( {1 - \gamma \beta } \right)}}\left[ {\frac{{\nu_{m} }}{{\left( {1 - \gamma \beta } \right)}} + b} \right] + b\left[ {\frac{{\nu_{m} }}{{\left( {1 - \gamma \beta } \right)}} - b} \right]}}$$

where *P* represents the pressure [MPa], *R* denotes the universal gas constant [MPa.m^3^/kmol/K], *T* is the absolute temperature [K], *ν*_*m*_ is the molar volume [m^3^/kmol], *a* and *b* represent attraction [MPa.(m^3^/kmol)^2^], and van der Waals volume terms [m^3^/kmol], respectively, *γ* is the dimensionless radius, and *β* indicates the reduced adsorption density. The values *a* and *b* are calculated as follows for the pure components^65,66^:

6 $$a = a_{c} \alpha$$

7 $$\alpha = \left[ {1 + m\left( {1 - T_{r}^{0.5} } \right)} \right]^{2}$$

8 $$\omega < 0.5$$

$$m = 0.37464 + 1.54222\omega - 0.26992\omega^{2}$$

9 $$\omega \ge 0.5$$

$$m = 0.3796 + 1.485\omega - 0.1644\omega^{2} + 0.01666\omega^{3}$$

10 $$a_{c} = \frac{{0.45724R^{2} T_{c}^{2} }}{{P_{c} }}$$

11 $$b = \frac{{0.07780RT_{c} }}{{P_{c} }}(1 - \gamma \beta )$$

The acentric factor, critical temperature [K], and critical pressure [MPa] are denoted by the letters *ω*, *T*_*c*_, and *P*_*c*_ in the above formulas.

The above equations also apply to mixtures using mixing rules^66^:

12 $$a_{mix} = \sum\limits_{i} {\sum\limits_{j} {x_{i} x_{j} a_{ij} } }$$

13 $$a_{ij} = \sqrt {a_{i} a_{j} } (1 - k_{ij} )$$

14 $$b_{mix} = \sum\limits_{i} {x_{i} b_{i} }$$

where *x*_*i*_ and *x*_*j*_ are mole fractions of the *i*th and *j*th components, respectively, and *k*_*ij*_ represents the binary interaction coefficient*.γ* and *β* can be computed in the following way^65^:

15 $$\gamma = \frac{2\delta }{{R_{p} }} - \left( {\frac{\delta }{{R_{p} }}} \right)^{2}$$

16 $$\beta = 0.6794\frac{{\left( {{{\sigma_{LJ} } \mathord{\left/ {\vphantom {{\sigma_{LJ} } {R_{p} }}} \right. \kern-0pt} {R_{p} }}} \right)^{0.7878} }}{{2\delta /R_{p} - (\delta /R_{p} )^{2} }}$$

In Eqs. (15 and 16), *R*_*p*_ denotes the pore radius [nm], *δ* and *σ*_*LJ*_ show the adsorption thickness [nm] and Lennard−Jones size parameter [nm], respectively, and are determined as follows^67^:

17 $$\sigma_{LJ} = 0.244\sqrt[3]{{T_{c} /P_{c} }}$$

18 $$\delta = \frac{{m_{ads} }}{{\ln (R_{p} /\sigma_{LJ} )}} + n_{ads} \left( {\frac{{\sigma_{LJ} }}{{R_{p} }}} \right)$$

19 $$m_{ads} = - 8.3140 \times 10^{ - 14} MW^{2} + 2.0475 \times 10^{ - 11} MW + 3.0886 \times 10^{ - 11}$$

20 $$n_{ads} = - 6.3565 \times 10^{ - 14} MW^{2} + 3.1550 \times 10^{ - 11} MW - 5.8538 \times 10^{ - 10}$$

The calculation of the fugacity coefficient for the *i*th component in the PR EoS is performed using Eq. (21)^66^:

21 $$\ln \varphi_{i}^{PR} = - \ln (Z - B) + \frac{{b_{i} }}{{b_{mix} }}(Z - 1) + \frac{A}{2\sqrt 2 B}\left( {\frac{{2\sum\nolimits_{J = 1}^{N} {x_{j} a_{ij} } }}{{a_{mix} }} - \frac{{b_{i} }}{{b_{mix} }}} \right)\ln \left[ {\frac{Z + (1 + \sqrt 2 )B}{{Z + (1 - \sqrt 2 )B}}} \right]$$

where *N* denotes the total number of components, and *A* and *B* are as follows^66^:

22 $$A = \frac{{a_{mix} P}}{{(RT)^{2} }}$$

23 $$B = \frac{{b_{mix} P}}{RT}$$

In Eq. (21), *Z* is the real and positive root of the following cubic equation^66^:

24 $$Z^{3} - (1 - B)Z^{2} + (A - 2B - 3B^{2} )Z - (AB - B^{2} - B^{3} ) = 0$$

### Cubic-Plus-Association (CPA) *EoS*

The CPA EoS has been successfully used to model the phase behavior of systems involving association components by a limited number of researchers^68^. CPA is an equation in which the simplicity of cubic equations in the physical part and the concepts of perturbation theory in the association part are combined. The resulting equation is not cubic relative to the volume and has five parameters for the pure components. Three of these parameters (*a, b, α*) belong to the physical and cubic part, and the remaining two parameters (*ε, β*_*CPA*_) are related to the association part and are specific to polar components. These values are obtained by fitting the vapor pressure and saturation fluid density data. But for non-associative components, it is more common to calculate them through critical properties and the acentric factor^68–70^. The general form of the CPA EoS is as follows^71^:

25 $$P = {\text{cubic EoS}} - \frac{1}{2}\frac{RT}{{\nu_{m} }}(1 + \rho \frac{\partial \ln g}{{\partial \rho }})\sum\limits_{i} {x_{i} \sum\limits_{{A_{i} }} {(1 - X^{{A_{i} }} )} }$$

where *ρ* is the molar density [kmol/m^3^], *g* is the radial distribution function, and *X*^*Ai*^ represents the fraction of sites A on molecule *i* that do not form bonds with other active sites^71^:

26 $$X^{{A_{i} }} = \frac{1}{{1 + \rho \sum\limits_{j} {x_{j} \sum\limits_{{B_{j} }} {X^{{B_{j} }} } \Delta^{{A_{i} B_{j} }} } }}$$

where Δ^*AiBj*^ is the association strength between site A of the *i*th molecule and site B of the *j*th molecule and is expressed by the following relation^71^:

27 $$\Delta^{{A_{i} B_{j} }} = gb_{ij} \beta_{CPA}^{{A_{i} B_{j} }} \left[ {\exp (\frac{{\varepsilon^{{A_{i} B_{j} }} }}{RT}) - 1} \right]$$

Elliott, et al.^72^ proposed a simple relation to computing the radial distribution function as follows:

28 $$g = \frac{1}{1 - 1.9\eta }$$

29 $$\eta = \frac{1}{4}b_{mix} \rho$$

*ε*^*AiBj*^ and *β*_*CPA*_^*AiBj*^ are association energy [J/kmol] and the association volume [m^3^/kmol], respectively. Equation (30)is a definition of the CPA EoS in terms of compressibility factor^71^:

30 $$Z^{CPA} = Z^{cub} + Z^{ass}$$

31 $$Z^{ass} = - \frac{1}{2}\left( {1 + \rho \frac{\partial \ln g}{{\partial \rho }}} \right)\sum\limits_{i} {x_{i} } \sum\limits_{{A_{i} }} {(1 - X^{{A_{i} }} )}$$

Equation (30)shows that the compressibility factor of the CPA EoS (*Z*^*CPA*^) can be calculated by adding this value for the cubic equation and the association part. These values are indicated by *Z*^*cub*^ and *Z*^*ass*^, respectively. The compressibility factor of the association part is calculated using Eq. (31) As discussed in the previous sections, the CPA EoS consists of a combination of a cubic equation and the association term. Therefore, to calculate its fugacity coefficient, it is necessary to add this value for both physical and association parts^71^:

32 $$\ln \varphi_{i} = \ln \varphi_{i}^{cub} + \ln \varphi_{i}^{ass}$$

The fugacity coefficient in the physical part is calculated by Eq. (21)and in the association part is as follows^71^:

33 $$\ln \varphi_{i}^{ass} = \sum\limits_{{A_{i} }} {\ln X^{{A_{i} }} } - \frac{{\rho b_{i} }}{8g}\frac{\partial g}{{\partial \eta }}\sum\limits_{i = 1}^{N} {x_{i} } \sum\limits_{{A_{i} }} {(1 - X^{{A_{i} }} )}$$

The compressibility factor is determined through trial and error whenever phase equilibrium calculations are conducted employing the CPA EoS. The compressibility factor in the association part of the CPA EoS is dependent on density and *X*^*Ai*^, as shown in Eq. (31). *X*^*Ai*^, on the other side, is computed employing density, as shown in Eq. (26). Therefore, to calculate the *Z*^*CPA*^ in each phase, the density of that phase must first be estimated. For the vapor phase, this value can be regarded as equal to the ideal gas density (*P/RT*), while for the liquid phase, it can be taken as equal to *b*_*mix*_. *X*^*Ai*^ is then calculated using density, and *Z*^*CPA*^ is obtained by substituting *X*^*Ai*^ and *ρ*. Using the new compressibility factor, the next estimation for the density is obtained, and this process continues until the desired error value is reached.

In this research, based on the model presented by Li and Firoozabadi^63,64^, the PR EoS has been employed in the cubic part of the CPA EoS. Additionally, the association energy and volume, the binary interaction coefficients, and mixing rules have been chosen based on the values suggested by them. SARA analysis shows that the oil sample utilized in this study comprises 5.2 wt% of asphaltene. For this reason, PVTp software was used to execute oil splitting based on the asphaltene weight percent. Then, according to Li and Firoozabadi^63,64^, the oil components were characterized by considering the pure components (N_2_, CO_2_, H_2_S, C_1_, C_2_, C_3_, iC_4_, nC_4_, iC_5_, and nC_5_), the pseudo-hydrocarbon components (C_6_-C_11_), and the hydrocarbon residue (C_12+_). The hydrocarbon residue is further divided into the heavy component and asphaltene, and the association between the asphaltene and heavy component was taken into account in the CPA EoS. The SARA analysis and splitting results are shown in Tables 2 and 4, respectively.

**Table 4:** Splitting results of the oil sample.

Components	Mole%	MW	Tc [K]	Pc [kPa]	Acentric factor	Parachor
N_2_	0.13	28.01	125.87	3392.37	0.039	60.40
CO_2_	0.15	44.01	304.09	7397.76	0.239	78.00
H_2_S	0	34.08	373.53	8963.00	0.0827	100.00
C_1_	33.90	16.04	190.64	4640.70	0.011	70.00
C_2_	8.42	30.10	305.26	4883.88	0.099	115.00
C_3_	8.69	44.10	369.82	4256.68	0.153	155.00
iC_4_	1.98	58.10	407.98	3647.71	0.183	181.50
nC_4_	2.81	58.10	424.98	3796.66	0.199	200.00
iC_5_	0.98	72.20	460.82	3330.56	0.227	225.00
nC_5_	1.16	72.20	469.59	3375.15	0.251	245.00
C_6_	1.78	86.20	507.65	3031.66	0.299	282.50
C_7_	2.15	99.50	539.98	2736.80	0.349	327.50
C_8_	4.39	112.00	569.26	2496.66	0.398	370.00
C_9_	2.21	125.00	594.82	2281.85	0.445	415.00
C_10_	1.58	137.00	618.71	2109.59	0.489	462.50
C_11_	1.04	149.00	639.82	1944.43	0.535	505.00
Heavy component	27.72	411.60	922.64	1323.31	0.943	1085.92
Asphaltene	0.91	896.39	1043.22	1227.31	1.115	3670.56

### Adsorption

Due to the presence of asphaltene in the studied oil sample and the predominance of asphaltene adsorption over other components, the adsorption effect was considered only for asphaltene. The molecular weight of the adsorbed component is required to determine the adsorption thickness according to Eq. (18) that proposed by Zhang, et al.^67^. The high molecular weight of asphaltene causes the values of *m*_*ads*_ and *n*_*ads*_ to be in the range whose values remain almost constant. Therefore, to calculate the thickness of asphaltene adsorption, the values of *m*_*ads*_ and *n*_*ads*_ were considered 1e9 and 4e9, respectively.

### Critical properties with confinement effect

Critical properties, including critical pressure and temperature, are influenced by the confinement effects. Since the critical properties are the primary inputs of EoSs for phase behavior calculations, each change in them can affect the performance of EoS. Zhang and Gu^8^ and Teklu, et al.^24^ found that these properties decreased in the porous media, and as the pore radius decreased, the deviation of the critical properties from the bulk phase increased. The reason for this phenomenon is that by decreasing the radius, fewer molecules are entrapped inside a pore, which causes the loss of fluid continuity and causes the critical properties to deviate from the bulk phase. In other word, when a fluid is examined in the bulk phase, it is a continuous phase with constant properties. However, as the scale of observation decreases towards the molecular level, the fluid loses its continuity and its properties begins to fluctuate. Nanopores have a limited capacity to accommodate only a few molecules, which leads to loss of fluid continuity and deviation from bulk properties^24^. Song, et al.^65^ presented the following equations to determine the critical properties in porous media:

34 $$\Delta P_{c} = \frac{{P_{c} - P_{cm} }}{{P_{c} }} = 1 - (1 - \gamma \beta )^{2} = 1.3588(\sigma_{LJ} /R_{p} )^{0.7878} - 0.4616(\sigma_{LJ} /R_{p} )^{1.3588}$$

35 $$\Delta T_{c} = \frac{{T_{c} - T_{cm} }}{{T_{c} }} = \gamma \beta = 0.6794(\sigma_{LJ} /R_{p} )^{o.7878}$$

Δ*T*_*c*_ and Δ*P*_*c*_ are the critical temperature and pressure shifts caused by confinement, respectively; *P*_*cm*_ and *P*_*c*_ are critical pressures [MPa] in nanopores and bulk phase, respectively; and *T*_*cm*_ and *T*_*c*_ are critical temperatures [K] in nanopores and bulk phase, respectively.

### Phase equilibrium calculations

Equilibrium is achieved between the vapor and liquid phases when the fugacity of all components in all phases is equal. So we have^66^:

36 $$x_{i} \varphi_{i}^{L} \left( {x,T,P^{L} } \right)P^{L} = y_{i} \varphi_{i}^{V} \left( {y,T,P^{V} } \right)P^{V}$$

where *x* and *y* represent the mole fractions of the liquid and vapor phases, respectively, and *φ* is the fugacity coefficient. The superscripts *L* and *V* refer to the liquid and vapor phases, respectively. Fugacity coefficients can be obtained using Eqs. (21 and 33) We need to solve the Rachford–Rice equations to determine the mole fraction of the components^73^:

37 $$\sum\limits_{i = 1}^{{N_{c} }} {\frac{{(K_{i} - 1)z_{i} }}{{1 + n_{\nu } (K_{i} - 1)}}} = 0$$

In Eq. (37), *n*_*v*_ is the number of moles of the vapor phase, and *z*_*i*_ is the total mole fraction of the *i*th component. *K*_*i*_ is the equilibrium constant of the *i*th component and is defined as *y*_*i*_*/x*_*i*_. In two-phase equilibrium calculations, the Wilson equation^66^ provides an initial guess of the equilibrium constant:

38 $$K_{i} = (P_{ci} /P)\exp \left[ {5.37(1 + \omega_{i} )(1 - T_{ci} /T)} \right]$$

After solving the Eq. (37), by the Newton method, if the fugacity coefficients of the components are not equal in all phases, we need a new assumption for the equilibrium constant:

39 $$K_{i}^{(n + 1)} = \frac{{f_{i}^{L(n)} }}{{f_{i}^{V(n)} }}K_{i}^{(n)}$$

where *f* denotes fugacity and *n* is the iteration number. In a porous medium, due to the curvature of the liquid–vapor interface, there is a pressure difference between these two phases called capillary pressure [MPa], which is as follows:

40 $$P_{cap} = P^{V} - P^{L}$$

The Young_Laplace equation for calculating capillary pressure is:

41 $$P_{cap} = \frac{2\sigma \cos \theta }{{R_{e} }}$$

*σ* is interfacial tension [mN/m], *θ* is contact angle, and *R*_*e*_ is effective pore radius^48^ [nm].

42 $$R_{e} = R_{p} - \delta$$

It is possible to figure out the interfacial tension using the following equation^48^:

43 $$\sigma = \frac{{\sigma_{\infty } }}{{1 + 2\frac{\delta }{{R_{e} }}}}$$

The interfacial tension, *σ*_*∞*_, in the flat condition may be computed using the parachor model developed by Macleod^74^ as follows:

44 $$\sigma_{\infty } = \left[ {\sum\limits_{i = 1}^{{N_{c} }} {\chi_{i} (x_{i} \rho^{L} (T) - y_{i} \rho^{V} (T))} } \right]^{4}$$

**Figure 1:**
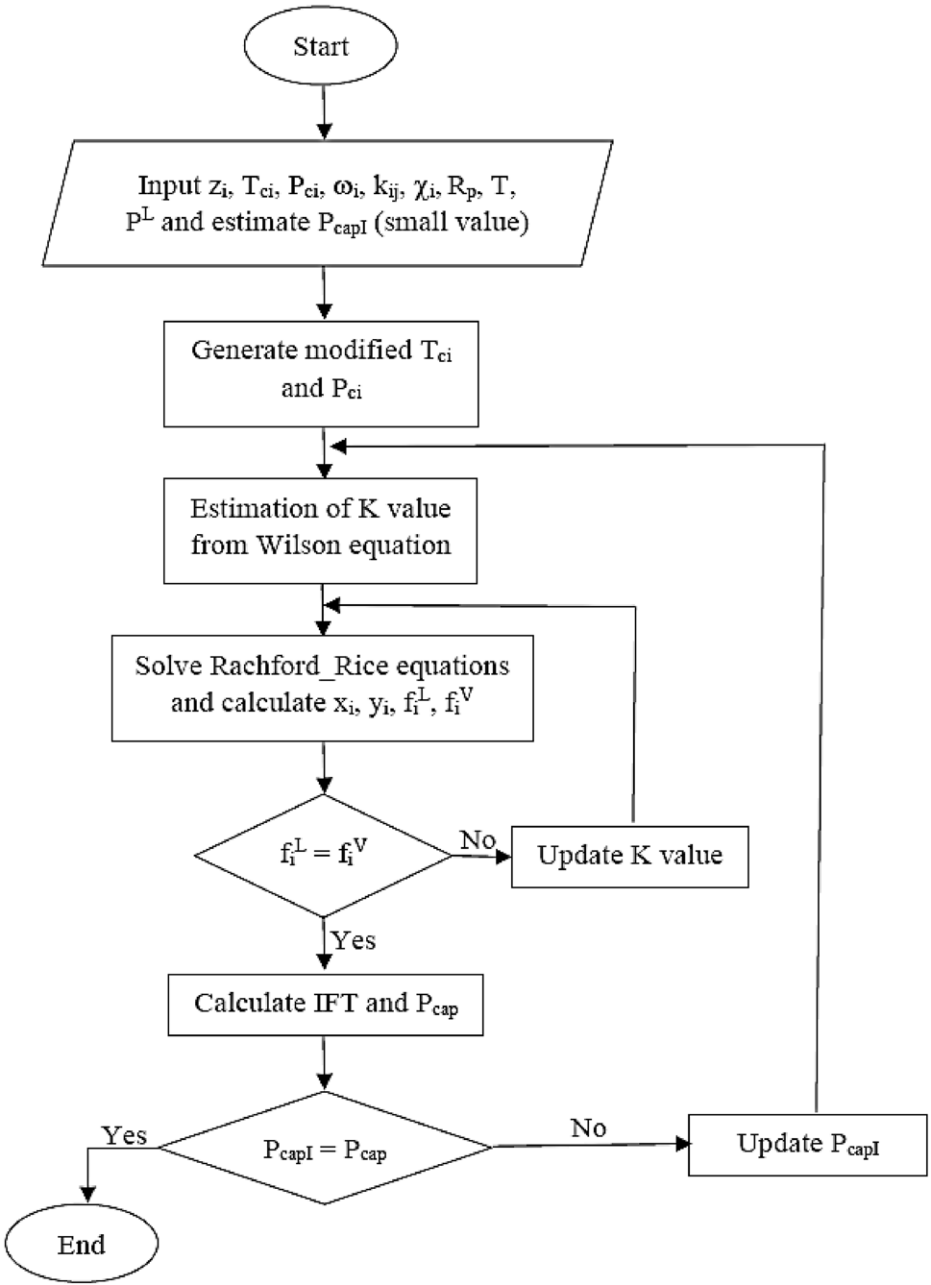
Flowchart of flash calculation in the presence of capillary pressure.

Molar densities of liquid and vapor phases are represented by *ρ*^*L*^ and *ρ*^*V*^, and the parachor is shown by *χ*. In this study, we used the suggested Song method to carry out flash calculations in the presence of capillary pressure^48,49^. The procedure is shown in Fig. 1. It is worth mentioning that the three-phase equilibrium calculations (between oil, vapor, and asphaltene phases) were carried out by the same algorithm using the CPA EoS, with the exception that the phase stability analysis^75^ was utilized for the initial approximation of the equilibrium constant between the oil phase and asphaltene.

### Calculations of the minimum miscibility pressure

**Figure 2:**
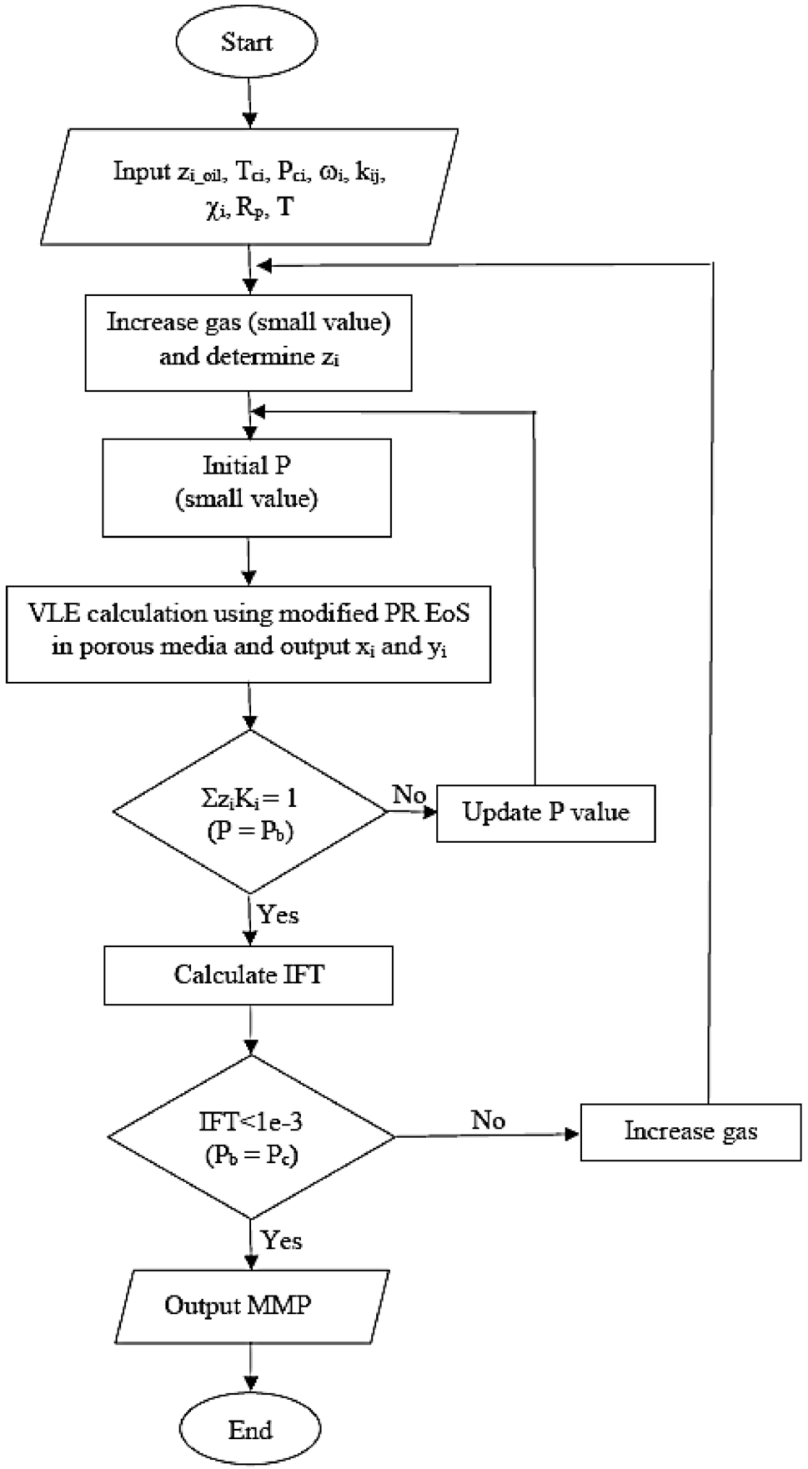
Flowchart of MMP calculation.

The VIT algorithm of Song was used in this paper to calculate the MMP^48,49^. Figure 2 illustrates the flowchart to compute the MMP.

This algorithm is inspired by Orr Jr and Jessen^76^ to calculate the MMP of the oil-CO_2_ system_,_ where the MMP is the minimum pressure at which oil and gas can be miscible in any proportion. We successfully used this method for our multicomponent gas samples. This is while most research has investigated single-component gases, especially CO_2_. Miscibility is achieved when the vapor and liquid phase properties are equal. In this way, the interface between the two phases is eliminated, and the fluid becomes a single phase. While approaching the critical point of a mixture, we notice a gradual convergence of the properties of both the liquid and vapor phases. Therefore, it may be argued that the miscibility range of oil and gas happens approximately around the critical point of their mixture. It should be noted that the MMP calculated in this way gives first contact miscible (FCM) MMP, which is obtained when the interfacial tension is very close to zero. The algorithm in Fig. 1 is used to calculate IFT at pressures below MMP. All coding and calculations regarding Figs. 1 and 2 were implemented in MATLAB R2021b.

## Results and discussion

In this section, we first present the results of the experimental measurements on the MMP between an asphaltenic oil and enriched natural gas samples, as well as the MMP calculations using the PR and CPA EoSs. Table S1 (Supplementary Material) shows the molar composition of the gas samples. VIT test data were available for the "Test Gas". We validate the developed codes and the calculation procedures using the test gas data. After that, the effects of important factors on the calculated MMP are investigated using other gas samples.

### Model validation

**Figure3:**
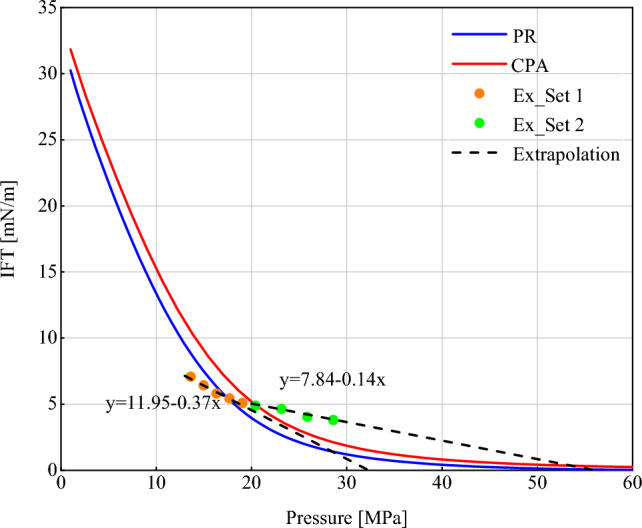
VIT test data and modeling results.

The results of the codes were compared with the VIT test data to evaluate the performance of the proposed model. VIT predicts the miscibility by calculating the interfacial tension between injected gas and an oil sample. In this efficient, reproducible, and less expensive method, MMP is calculated by extrapolation of the IFT curve until it reaches zero^47,77^. Figure 3 shows the experimental data points as well as the model outputs. Since the VIT test was not performed in porous media, the value of *R*_*p*_ was assumed to be equal to infinity (∞). The test temperature was 294 K. A closer look at the experimental results in Fig. 3 reveals that the data have two different trends that are indicated by two colors in the figure. At low pressures (Set 1), IFT decreases at a relatively high rate. If this trend continues, the MMP can be estimated by extrapolating the data until zero IFT is reached. But the rate of IFT reduction slows down with increasing pressure (Set 2). Therefore we can consider the value of 7.84/0.14 = 56 MPa as MMP. As it is obvious the calculated IFT at this point using the model is about zero. As shown in Fig. 3, the PR EoS has a more accurate prediction in the first part, while the CPA EoS performs better in the second part. These multiple slopes in the VIT data can be attributed to the presence of asphaltene in the oil sample. In asphaltenic oils, the thermodynamic equilibrium is disturbed by the gas dissolution process, and the asphaltene molecules are separated from the bulk phase. Due to their polarity, these molecules act as surfactants and come into the oil–gas interface and change the trend of IFT reduction So, the data breakpoint can be considered as the asphaltene onset point. This is why the CPA EoS provides a more accurate prediction in the second part. Table 5 shows the AAD% calculated according to Eq. (45) for both equations in both intervals.

45 $$AAD\% = 100 \times \frac{{\left| {IFT_{test} - IFT_{model} } \right|}}{{IFT_{test} }}$$

where *AAD*% is the percentage of average absolute deviation, and *IFT*_*test*_ and *IFT*_*model*_ are IFTs obtained by the VIT test and calculated by the model, respectively. (refer to Section "Comparing the performance of the PR and CPA EoSs" and the Supplementary Material for more information about distinct slopes of IFT curve).

**Table 5:** AAD% of PR and CPA EoS before and after the asphaltene onset pressure.

EoS	AAD%		
	Set 1	Set 2	Total
PR	12.97	45.40	27.38
CPA	32.16	24.43	28.73

**Figure 4:**
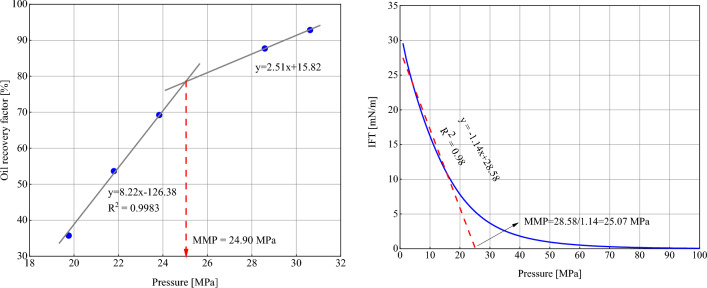
Slim tube data and CPA modeling results.

It is important to note that our model is designed for use in porous media, and there exists no experimental test to measure IFT in porous media. For this reason, the results of the model have been forced to compare with the VIT test data. It is worth noting that based on the algorithm presented in Fig. 2, the percentage of oil and gas combination is automatically calculated by the code to form the critical mixture and reach the first contact miscible pressure. It is well known that in the VIT test, a drop of oil is contained within a gas chamber and the overall gas-oil composition is not measurable. If we were able to measure this composition, the outcome of our model would be more congruent with the experiment. Despite our efforts to ensure consistency, limitations in the experimental conditions prevent the perfect comparison of the results of our model with the VIT test. Nevertheless, in the absence of alternative measurements, this remains the closest method for modeling the IFT behavior. The dependence of the VIT results on the overall composition of the gas-oil mixture has been investigated by some researchers^76^. On the other hand, the slim tube test can take into account the effects of the porous media on MMP, but its output shows the oil recovery factor versus pressure, which is not directly comparable with the output of the model. The results of the model can be indirectly compared with the slim tube test. In such a way that the MMP obtained from the slim tube test (which is the multiple contact miscibility pressure) is compared with the first part of the model. Figure 4 shows the results of the model and the MMP obtained from the slim tube test. The test temperature, porosity and permeability of the slim tube are 372 K, 29% and 3600 mD, respectively (*R*_*p*_ has been considered 1e9 × √(3600e-15/0.29) nm as it will be discussed in Section "Effect of injected gas" with more detail). As can be seen, the output of the model has a difference of about 0.7% with the test result. This way, we reduced the uncertainty of the model and led the results to be compared with the porous medium.

### Effect of pore radius

**Figure 5:**
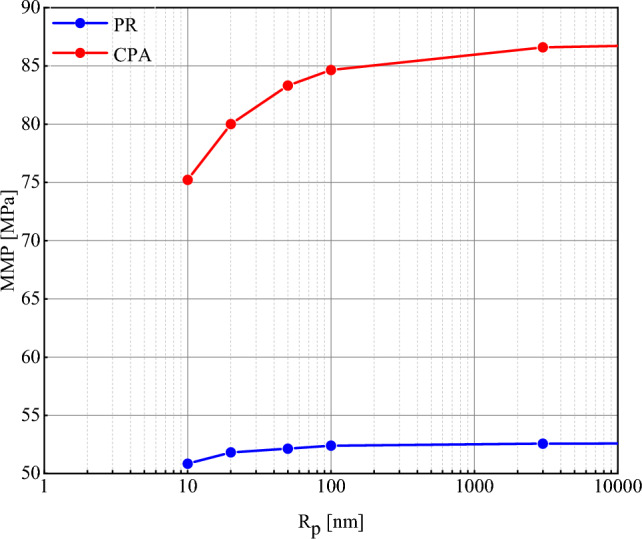
Investigation of the effect of pore radius on MMP of oil_NGL230 mixture (T = 372 K).

The influence of the pore radius on the MMP value of oil_ NGL230, determined using both the PR and CPA EoSs, is depicted in Fig. 5. As can be seen, for both equations, the MMP increases with increasing radius, but this trend slows down at R_p_ greater than 100 nm, and the MMP is almost constant in this range. At radius smaller than 100 nm, the effect of capillary pressure is impressive and non-negligible. But, after that with increasing pore radius, capillary pressure approaches to zero and the fluid behavior is as same as bulk phase. Therefore, it can be said that MMP deceases under confinement effect and miscibility happens in lower pressures in porous media. To explain this behavior, the presence of a gas bubble within the bulk of the liquid phase is considered. Our goal is to raise the pressure to the MMP and dissolve the bubble in the liquid. In thermodynamics, this process is typically investigated in two stages, beginning with the gas bubble converting to a liquid and ending with the liquid dissolving in the liquid. The pressure required to turn a bubble into a liquid is its saturation pressure, which is called the dew point pressure. Among the various gas bubbles, the bubble with the lowest saturation pressure turns into a liquid faster and has a lower MMP. However, it should be noted that the experimental saturation pressure is different from the saturation pressure in porous media where the interface is curved. This difference is expressed by the Kelvin equation^78^:

46 $$P_{\nu } = P^{sat} \times \exp \left[ { - \frac{2\sigma }{{rRT\rho }}} \right]$$

The Kelvin equation provides the saturation pressure (*P*_*v*_[MPa]) of a bubble as a function of the radius of curvature of the interface (*r* [nm])^79^. In Eq. 46, *P*^*sat*^ represents the saturation pressure [MPa] in the bulk phase, and *σ* is interfacial tension [mN/m]. Increasing the pore radius leads to an increase in *r* and consequently an increase in *P*_*v*_. Therefore, the gas bubble has a higher MMP at a larger radius and reaches miscibility later.

Molecule–molecule and molecule-pore wall distances are drastically reduced at a radius of less than 100 nm. In this situation, phase behavior is not only controlled by the interactions between fluid molecules but also by the interactions of fluid molecules-pore walls. At radii greater than 100 nm, the fluid gradually gets closer and closer to the bulk phase, and therefore the MMP remains almost constant after 100 nm. Because of this, it should be noted that the determination of MMP is more important in unconventional reservoirs such as shale reservoirs where the pore radius is very small. On the other hand, the smaller pore radius of a reservoir makes the injected gas reach miscibility at lower pressures, and gas injection is a more suitable option for EOR processes.

### Comparing the performance of the PR and CPA EoSs

**Figure 6:**
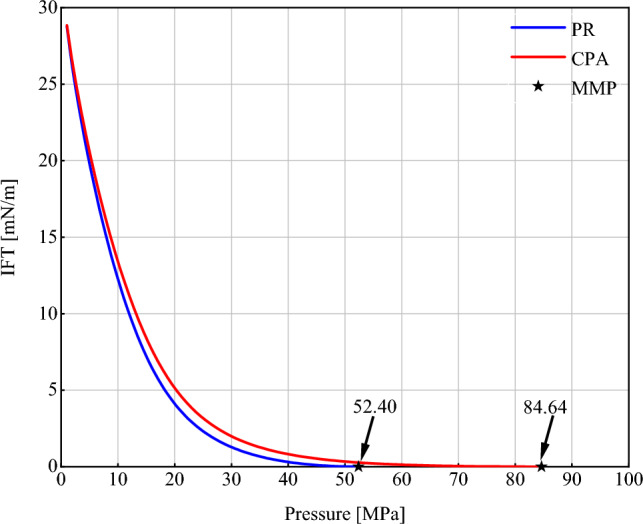
Comparing the results of PR and CPA EoS for oil_NGL230 mixture at *R*_*p*_ = 100 nm and *T* = 372K.

In the previous section, the PR and CPA EoSs were used to study the MMP alterations by increasing the pore radius. When looking at the findings properly, it can be seen that, at a given radius, the MMP calculated using the CPA EoS was higher than the MMP obtained from the PR EoS. Additionally, according to Fig. 5, the rate of MMP ascent with an increasing radius for the CPA EoS was higher than the PR EoS. This can be due to the presence of asphaltene in the studied oil sample and considering the precipitation phenomenon using the CPA EoS. Figure 6 shows the IFT reduction with increasing pressure for NGL230 at a radius of 100 nm and T = 372 K. Both curves have two different trends. In the first part of the curve and at low pressures, the slope of IFT reduction is high. In this portion, the reduction in IFT is due to the mass transfer between oil and gas. As a result of this exchange, the liquid and vapor phase properties converge, and the IFT reduction occurs more rapidly. Therefore, it is expected that by continuing this process until IFT reaches zero, the value of MCM pressure will be obtained. But in the second portion of the curve, the IFT reduction slope decreases. This reduction occurs for the CPA EoS at a slower rate than the PR EoS. To explain this phenomenon, it should be said that in the first section, the asphaltene molecules are suspended in the oil phase and form a homogeneous mixture. Gradually, as the gas dissolves in the oil, the asphaltene molecules lose their stability. Due to their polarity, they act as surfactants and begin to accumulate on the oil_gas interface and reduce the solubility of the gas in the oil. Thus, counteracting the two opposite factors (increasing gas dissolution due to increasing pressure and decreasing gas dissolution due to accumulation of asphaltene molecules on the interface) reduces the downward trend of IFT and delays the miscibility. If the accumulation of asphaltene molecules exceeds a threshold (covering more than 60% of the interface), the effect of asphaltene on the IFT reduction process is more significant, and the attainment of MMP is further delayed. This phenomenon has been experimentally investigated in previous research ^51–57^. Asphaltene precipitation on the gas-oil interface, has a huge impact on miscibility condition. These molecules, as a surface-active component, accumulate on the interface and greatly change the IFT reduction slope. In low pressure interval, gas solubility and mass transfer between oil and gas are dominant. But, after onset pressure, the accumulation of asphaltene molecule cause the reduction in solubility and delay miscibility. Therefore, in high pressure interval, IFT reduces with lower slope. As can be seen, the miscibility pressure with considering asphaltene presence is about 61% different from the model without asphaltene precipitation effect.

**Figure 7:**
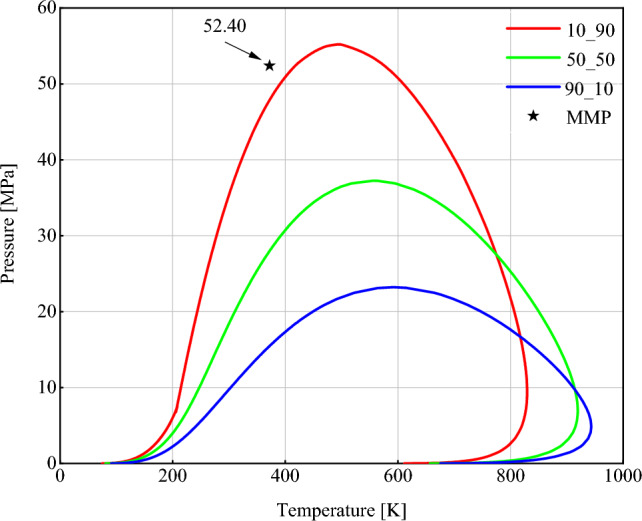
Phase envelope for different percentages of oil and gas and the corresponding MMP of their mixture using the PR EoS (10_90 refers to a mixture containing 10 mol% oil and 90 mol% gas).

Figure 7 shows the phase diagrams of oil and NGL230 mixtures with different mixing percentages. These diagrams are obtained using the PR EoS. As shown in Fig. 7, the location of the MMP is at a point within the single-phase region for all mixtures. As it is mentioned in Section "Calculations of the minimum miscibility pressure", algorithm used in this study measures the FCM pressure. First contact miscibility means oil and gas are miscible at the first contact and with any proportion. Therefore, it can be concluded that the used algorithm is able to predict the MMP correctly.

### Effect of injected gas

**Figure 8:**
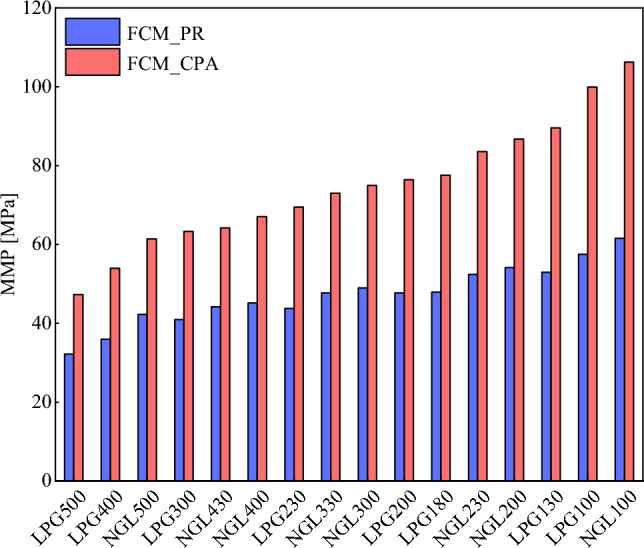
MMP estimated by the PR and CPA EoSs for different gas samples (*T* = 372 K, *R*_*p*_ = 1e9 × √(1e-15/0.08) nm).

Pore radius is a microscopic feature of reservoir rock, and assigning a unique pore radius for a rock may be impractical. Alternatively, porosity (*ϕ*) and permeability (*k*) in different sections of a reservoir (such as matrix and fractures) are widely accessible from the core analysis. The square root of the permeability to porosity ratio (√(*k*/*ϕ*)) is the Leverett's mean hydraulic radius and has widely been used as an approximation of the mean pore size of a rock^80^. The oil sample studied in this research was taken from a carbonate reservoir with a permeability of about 1 mD and a porosity of 8%. Consequently, we considered the *R*_*p*_ to be 1e9 × √(1e-15/0.08) nm and investigated the MMP of different gas samples. Figure 8 shows the MMP estimated by both equations. Referring to Table S1 (Supplementary Material), if we compare the composition of different gases, LPG100 and NGL100 are the lightest samples, and LPG500 is the heaviest. Enriching the injected gas and adding heavy components to it reduces the MMP. Therefore, the heaviest and lightest samples are expected to have the lowest and highest MMP, respectively. It is evident that the proposed models well predict this trend. This information makes it possible to optimize the selected gas before performing the gas injection processes by considering the MMP of each of the available samples and evaluating the equipment and economic conditions. It should also be noted that MMP can vary in different reservoir zones. For example, the MMP in the matrix will be less than this value for the fracture, and this point should be paid special attention to in the design of the gas injection processes.

### Effect of asphaltene type

As mentioned in Section "Comparing the performance of the PR and CPA EoSs", the presence of asphaltene in oil significantly affects IFT reduction and achieving miscibility. Different oils have different asphaltene molecules with different structures and interactions. This issue manifests itself in the form of a difference in asphaltene molecular weight. Now it is interesting to assess the influence of asphaltene type in terms of molecular weight on the MMP. Table 6 reports the calculated MMP using the CPA EoS by changing the molecular weight of asphaltene from 400 to 1200. Comparing the obtained values, it seems that the molecular weight of asphaltene has little impact on MMP, and the weight percentage of asphaltene in the oil controls the rate of IFT alterations.

**Table 6:** Effect of asphaltene type on MMP. (oil + NGL230, *R*_*p*_ = 100 nm, *T* = 273 K).

	Asphaltene MW		
	400	800	1200
MMP [MPa]	85.18	84.63	81.73

According to the abovementioned results, it can be said that both nanopores confinement and the asphaltene presence in oil significantly affect the phase behavior and reaching miscibility conditions in the porous medium. These changes are more evident in smaller pores. Therefore, it is vital to pay special attention to these differences in gas injection processes. Because changes in miscibility conditions can affect the reservoir production and economical evaluations. Additionally, asphaltene precipitation has a lot of technical drawbacks, such as pore blocking, and its prediction in porous media is of special importance.

## Conclusions

This study investigated the MMP between an asphaltenic crude oil and enriched natural gases considering both the nanopore confinement effect and asphaltene precipitation simultaneously. This is despite the fact that previous researches have considered only one of these two important factors. This is the first time that a model has been developed to include both confinement and asphaltene precipitation effects. The experimental measurements based on the VIT and slim tube methods were carried out to validate the proposed models. The impact of pore radius, type of injected gas, and asphaltene molecular weight on the MMP were also examined. The main results of this paper are summarized as follows:

- Confinement effect was more crucial when the pore radius was less than 100 nm. Therefore, the calculation of MMP in unconventional tight reservoirs such as shale reservoirs should be performed more accurately.
- Asphaltene presence delayed the miscibility by about 61% compared to model without asphaltene precipitation effect. Therefore, in modeling the phase behavior of asphaltenic oils and calculating their MMP, it is suggested to use association EoSs with more accurate predictions in the asphaltene precipitation range.
- MMP in various zones of a reservoir may have different values. This means that gas injection in unconventional and fractured reservoirs should be carried out by considering variations in MMP and modified EoSs can be used to optimize the selected gas before gas injection EOR processes.

Finally, it is worth noting that the study of confined fluid is of practical application in any field related to porous media such as carbon capture and storage (CCS), hydrogen storage, and enhanced oil recovery from shale reservoirs. Considering the ability of association EoSs in predicting asphaltene precipitation, it is possible to investigate the effect of injected gas on the amount of asphaltene precipitation in future studies. Also, the method used in this research combining the Song method^62^ can be applied for reservoir simulations to predict the effects of phase behavior changes due to nanopore effects on reservoir performance and production during the gas injection process. Future studies should consider a multifaceted approach to asphaltene precipitation and its impact on minimum miscibility pressure calculations, extending beyond the comparison of PR and CPA EoSs. The incorporation of empirical correlations, molecular dynamics simulations, and advanced thermodynamic models like PC-SAFT EOS is recommended to provide a more comprehensive understanding of asphaltene behavior under diverse reservoir conditions.

### Declaration of generative AI and AI-assisted technologies in the writing process

During the preparation of this work the authors used https://typeset.io/paraphraser in order to paraphrase. After using this tool/service, the authors reviewed and edited the content as needed and take full responsibility for the content of the publication. 

### Supplementary Information

Supplementary Information.

## Data Availability

The authors confirm that the data supporting the findings of this study are available in the article and in the supplementary information.

## Abbreviations

*A*: EoS parameter for compressibility factor calculation
*a*: Attractive term [MPa (m^3^/kmol)^2^]
*a*_*c*_: PR EoS parameter [MPa (m^3^/kmol)^2^]
*B*: EoS parameter for compressibility factor calculation
*b*: Van der Waals volume term (m^3^/kmol)
*f*: Fugacity
*g*: Radial distribution function
*K*_*i*_: Equilibrium constant of *i*th component
*k*: Permeability (mD)
*k*_*ij*_: Binary interaction coefficient
*MW*: Molecular weight (g/gmol)
*m*: PR EoS parameter
*m*_*ads*_: A function of MW for adsorption thickness calculation
*N*: Total number of components
*n*: Iteration number
*n*_*ads*_: A function of MW for adsorption thickness calculation
*n*_*v*_: Number of moles of the vapor phase
*P*: Pressure (MPa)
*P*_*c*_: Critical pressure in bulk phase (MPa)
*P*_*cap*_: Capillary pressure (MPa)
*P*_*cm*_: Critical pressure in nanopores (MPa)
*P*^*L*^: Liquid pressure (MPa)
*P*^*sat*^: Bubble saturation pressure in the bulk phase (MPa)
*P*_*sc*_: Gas pressure in standard condition
*P*^*V*^: Vapor pressure (MPa)
*P*_*v*_: Bubble saturation pressure (MPa)
*R*: Gas universal constant (MPa.m^3^/kmol/K)
*R*_*e*_: Effective pore radius (nm)
*R*_*p*_: Pore radius (nm)
*r*: Radius of curvature (nm)
*T*: Temperature (K)
*T*_*c*_: Critical temperature in bulk phase (K)
*T*_*cm*_: Critical temperature in nanopores (K)
*T*_*r*_: Reduced temperature
*T*_*sc*_: Gas temperature in standard condition
*V*: Volume
*V*_*sc*_: Gas volume in standard condition
*X*^*Ai*^: The fraction of sites A on molecule i that do not form bonds with other active sites
*x*_*i*_: Mole fraction of the *i*th component
*y*_*i*_: Mole fraction of the *i*th component in vapor phase
*Z*: Compressibility factor
*Z*_*sc*_: Gas compressibility factor in standard condition
*z*_*i*_: Total mole fraction of *i*th component

## Greek symbols

*α*: PR EoS parameter
*β*: Reduced adsorption density
*β*_*CPA*_: Association volume (m^3^/kmol)
*γ*: Dimensionless radius
*Δ*^*AiBj*^: Association strength
*δ*: Adsorption thickness (nm)
*ε*: Association energy (J/kmol)
*η*: CPA EoS parameter
*θ*: Contact angle
*ν*_*m*_: Molar volume (m^3^/kmol)
*ρ*: Molar density (kmol/m^3^)
*σ*: Interfacial tension (mN/m)
*σ*_*LJ*_: Lennard − Jones size parameter (nm)
*σ*_*∞*_: Interfacial tension in flat condition (mN/m)
$$\varnothing$$: Fugacity coefficient
*φ*: Porosity
*χ*: Parachor
*ω*: Acentric factor
