# Supplementary material for: Impact of nanopore confinement on phase behavior and enriched gas minimum miscibility pressure in asphaltenic tight oil reservoirs
Source: Sci Rep. 2024 Jun 11;14:13405. doi: 10.1038/s41598-024-64194-2 (PMC11167058; doi:10.1038/s41598-024-64194-2)
Supplement: Supplementary file 1 — Supplementary Information. [file 41598_2024_64194_MOESM1_ESM.docx]

**Impact of nanopore confinement on phase behavior and enriched gas minimum miscibility pressure in asphaltenic tight oil reservoirs**

Fatemeh Keyvani^a^, Ali Safaei^b,c^, Yousef Kazemzadeh^d^, Masoud Riazi^c,e*^, and Jafar Qajar^a,f*^

*^a^Department of Petroleum Engineering, School of Chemical and Petroleum Engineering, Shiraz University, 7193616511, Shiraz, Iran*

*^b^Fouman Faculty of Engineering, College of Engineering, University of Tehran, 4358139115, Tehran, Iran*

*^c^Enhanced Oil Recovery (EOR) Research Centre, IOR/EOR Research Institute, Shiraz University 7193616511, Shiraz, Iran*

*^d^Department of Petroleum Engineering, Faculty of Petroleum, Gas, and Petrochemical Engineering, Persian Gulf University, 7516913817, Bushehr, Iran*

*^e^School of Mining and Geoscience, Nazarbayev University, Kabanbay Batyr 53, 010000, Astana, Kazakhstan*

*^f^Department of Earth Sciences, Faculty of Geosciences, Utrecht University, 3584 CS Utrecht, The Netherlands*

^*^Corresponding authors:

Email addresses: jqajar@shirazu.ac.ir (J. Qajar), masoud.riazi@nu.edu.kz (M. Riazi)

**Supplementary Information**

**Background:**

Teklu, et al. ^1^, considering the effect of capillary pressure, developed a computational method that calculated the interfacial tension (IFT) between oil and gas according to the vanishing interfacial tension (VIT) experimental technique and hence calculated the MMP by continuing this trend until zero IFT was reached. Then, to evaluate the performance of their method, they calculated the MMP for different oil samples with different types of hydrocarbon and non-hydrocarbon gases and concluded that the MMP in the porous media is lower than in the bulk phase. In another study, Teklu, et al. ^2^ developed conventional VLE calculations considering the effect of capillary pressure and critical properties shift and then investigated the effect of confinement on bubble point, dew point, and IFT between oil and injected gas. They then calculated the MMP using both the multiple mixing cell (MMC) algorithm and their improved method. The results showed that the bubble point pressure, IFT, MMP, and lower dew point pressure decreased compared to the bulk phase, while the upper dew point pressure increased. A novel method based on the PC-SAFT EoS was developed by Wang, et al. ^3^ to estimate the MMP of the oil-CO_2_ system in nanopores and the bulk phase. Zhang et al. ^4,5^ first modified the Peng-Robinson (PR) EoS by considering the effects of capillary pressure and the shift of critical properties for use in the network of nanopores. They then coupled the modified PR EoS with the parachor model (parachor is one of the characteristics of components that is related to surface tension and introduced by Sugden ^6^) and developed a new method called the diminishing interface method (DIM) to calculate the MMP. In this method, the MMP is calculated using the derivative of the interfacial thickness with respect to the pressure. Using their proposed method, they obtained the MMP between different oil samples with CO_2_ and concluded that the MMP decreases in the porous media. They also found that the MMP was highly dependent on the composition of the initial oil and injected gas. Zhang, et al. ^7^ investigated the effect of capillary pressure on oil-CO_2_ MMP using three different methods: multiple mixing cells (MMC), method of characteristics (MOC), and the slim tube simulation method. They concluded that high capillary pressure altered the vapor and liquid phase compositions and changed the MMP. The corresponding changes in the MMP were dependent on the initial oil composition. Zhang, et al. ^8^ divided the existing empirical correlations for the MMP estimation into three categories: (1) temperature-dependent, (2) temperature and oil composition-dependent, and (3) temperature, oil composition, and gas composition-dependent. None of these correlations took into account the effect of pore structure. They presented a new empirical correlation for estimating the MMP of oil and gas. In this correlation, the MMP was calculated based on temperature, C_5+_ molecular weight, mole fraction ratios of volatile components to intermediate components, and pore radius. This empirical correlation accurately estimated the MMP of various oil and gas samples. They also found that the MMP remained constant at a radius greater than 100 nm and that the MMP decreased as the radius reduced to less than 100 nm. In another study, Mohammad, et al. ^9^, considering the effect of nanopore confinement, developed a correlation to estimate CO_2_-oil MMP and compared its results with CMG software. The correlation showed good agreement for live oil but larger deviation for dead oil. The results highlighted the importance of accounting for nanopore confinement effects when predicting MMP in tight reservoirs. Song, et al. ^10,11^ first proposed a model for performing VLE calculations based on the PR EoS. This model was able to take into account the effects of adsorption, the shift of critical properties, and capillary pressure. They then coupled their proposed model with the VIT algorithm and used it to calculate the MMP between Bakken oil and CO_2_. The results showed that the presence of nanopores reduced the difference between the liquid and vapor phases and led to IFT reduction. Therefore, by decreasing the pore radius, the MMP also decreases, and this trend is more important at a radius of less than 20 nm. Sun and Li ^12^ proposed a new algorithm based on the method proposed by Ahmadi and Johns ^13^ to calculate the MMP, which also considered the effects of pore size distribution, capillarity, and confinement. They applied the PR EoS with the volume shift proposed by Abudour, et al. ^14^ in their algorithm and used experimental data of Zhang and Gu ^15^ to tune the EoS and optimize the BIC (Binary Interaction Coefficient) values. They also coupled capillary pressure and shift of critical properties models with the EoS to account for the effect of nanopores on phase equilibrium calculations. Using this proposed model, they investigated the effect of temperature and pore size on the MMP of oil-CO_2_. The results showed that considering the volume shift, the error in estimating IFT between liquid and gas is reduced. Also, reducing the pore radius reduces the MMP, but at a radius greater than 10 nm, the MMP value remains almost constant. They found that for each radius, a maximum MMP is obtained with increasing temperature, which decreases with decreasing radius. Sun and Li ^16^ used the method proposed by Metcalfe, et al. ^17^ and the minimum tie-line length criterion to calculate the MMP between oil and gas. VLE calculations were performed using the volume-translated PR EoS, considering the effect of capillary pressure and the shift of critical properties. They then studied the effect of temperature, pore radius, and impurities on the MMP value. The results showed that MMP decreases in confined spaces. Also, at a given radius, the MMP first increases and then decreases with increasing temperature (there is a maximum MMP for each radius), and adding impurities to the injected gas increases the MMP.

As a popular tool, molecular simulation has recently become an alternative method for investigating reservoir fluid and rock properties and their interactions at a molecular scale and replaces costly or hazardous experiments with "computer experiments" ^18-21^. Using this technology, Peng et al. ^22^ and Cui et al. ^23^ have attempted to investigate the MMP between oil and CO_2_. They used the VIT technique and studied miscibility conditions by calculating IFT in the interface of oil and gas. Although these studies offer valuable insight into molecular-scale phenomena, they only considered molecule-molecule interactions and neglected molecule-wall interactions.

Asphaltene precipitation is an important phenomenon that occurs in gas injection processes. By injecting gas, the phase equilibrium of the reservoir fluid is disturbed, and some of its components, such as asphaltene, are separated from the bulk phase. Asphaltene, as a surface-active molecule, can accumulate on the interface of oil and gas and affect the miscibility process. In the abovementioned studies, MMP has been investigated considering the effect of confinement or in a molecular scale, but the effect of asphaltene precipitation has not been considered. Some researchers have tried to investigate the impact of asphaltene precipitation on IFT reduction and reaching the miscibility using laboratory experiments ^24-30^. Results showed that three distinct slopes can be identified in the decrease of IFT between oil and gas. IFT reduction during the low-pressure range may be primarily attributed to greater mass transfer between the gas and oil phases (first slope). As pressure increases, a greater amount of gas dissolves in the oil phase, causing the oil phase to swell and the gas-oil IFT to decrease. However, the degree of IFT reduction during the high-pressure range is less than the low-pressure interval (second slope). During the high-pressure range, although gas dissolution leads to a decrease in IFT, the accumulation of asphaltene at the interface of the oil phase and gas occurs, ultimately increasing IFT (third slope). The IFT data trend is significantly amplified when the surface coverage of the particles exceeds a threshold value (e.g., + 60% surface coverage). In these studies, researchers have especially focused on the effect of asphaltene precipitation on the IFT reduction trend and miscibility process, but the confinement effect has not been considered.

**Experimental techniques:**

*Vanishing interfacial tension (VIT) test*

A general schematic of the setup (IFT700, Vinci Technologies, France) used in this investigation to measure the IFT between the live oil and the injected gas is shown in Figure S.1. The main part of this setup is a high-pressure visual cell with a volume of 20 cc. On either side of the cell are two sight glasses, one facing the camera and the other with a backlight to provide enough light to take images from inside the cell. The images received by the camera are also analyzed by the software of the IFT700 apparatus (axisymmetric drop shape analysis (ADSA)). To perform the test, it is first necessary to vacuum the setup lines and cell by using a vacuum pump and through valve 3 (refer to Figure S.1). Then, the cell should be filled with the bulk fluid (gas) and reach the desired pressure of the test. After the cell pressure reaches the desired pressure, a drop of the live oil is injected into the cell through valve 1 using a piston-cylinder system containing the live oil, which is connected to a high-pressure syringe pump.

It should be noted that to form the oil drop, a steel needle with an outer diameter of 1.58 mm was used. Valve 1 must be closed after the droplet has formed so that only the volume of the injected oil droplet is in contact with the gas in the cell. After the drop is formed, sufficient time is given to the system to reach thermodynamic equilibrium. Then, the final volume of the droplet and the dimensionless Bond number remain constant. These two values are recorded and saved automatically by the IFT software. The amount of oil-gas interfacial tension is also measured according to the initial oil and gas density values during the test time. The mentioned procedure is repeated for each pressure step.


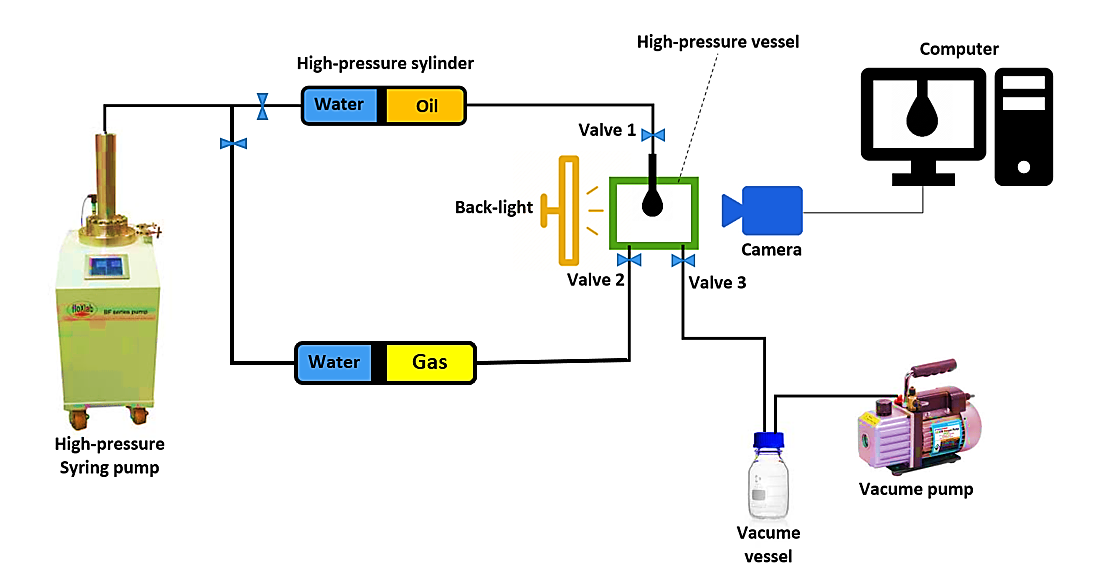


**Figure S.1.** The general schematic of the interfacial tension apparatus (measurement precision=±0.2 mN/m)

*Slim tube test*

To conduct the slim tube displacement test in this study, the slim tube apparatus (STT1000, Vinci Technologies, France) depicted in Figure S.2 was utilized. The test was carried out at a temperature of 210℉ (372 K) and involved five pressure steps. Further details of the test procedure can be found elsewhere ^31^. During each pressure step, gas was introduced at a flow rate of 0.08 cc/min. The experiment maintained a consistent injection flow rate of 0.08 cc/min, with gas injection spanning 1.2 pore volumes of the slim tube. The specifications of the slim tube utilized in this investigation are outlined in Table S.1.


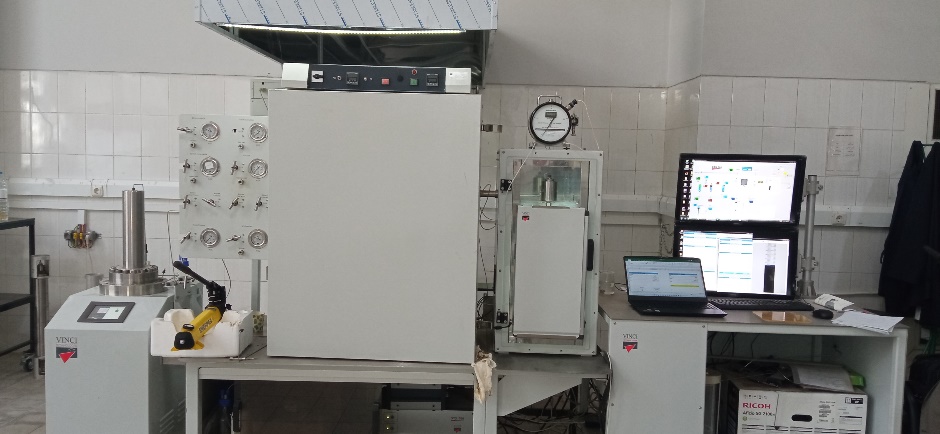


**Figure S.2.** General view of slim tube apparatus.

**Table S.1.** The properties of the slim tube in this study.

| Properties | Values or specifications |
| --- | --- |
| ID of the slim tube (mm) | 4.00 |
| Length of the slim tube (ft) | 40.00 |
| Slim-tube material | 316 stainless steel |
| Pore volume (cc) | 100.6009 |
| Permeability (mD) | 3600 |
| Porosity (%) | 29 |

**Table S.2.** Molar composition of gas samples.

| LPG500 | 5.47 | 0.91 | 0.00 | 46.38 | 10.31 | 15.67 | 3.37 | 8.16 | 2.78 | 2.77 | 2.76 | 0.84 | 0.48 | 0.10 |
| --- | --- | --- | --- | --- | --- | --- | --- | --- | --- | --- | --- | --- | --- | --- |
| LPG400 | 5.98 | 1.00 | 0.00 | 50.60 | 9.26 | 13.79 | 2.99 | 7.20 | 2.50 | 2.47 | 2.66 | 0.92 | 0.53 | 0.11 |
| LPG300 | 6.59 | 1.10 | 0.00 | 55.69 | 8.00 | 11.52 | 2.53 | 6.04 | 2.17 | 2.10 | 2.54 | 1.02 | 0.58 | 0.12 |
| LPG230 | 7.21 | 1.20 | 0.00 | 60.51 | 1.48 | 6.47 | 9.28 | 9.78 | 0.44 | 0.27 | 1.47 | 1.11 | 0.64 | 0.13 |
| LPG200 | 7.35 | 1.23 | 0.00 | 61.94 | 6.45 | 8.73 | 1.97 | 4.62 | 1.76 | 1.66 | 2.39 | 1.13 | 0.65 | 0.13 |
| LPG180 | 7.61 | 1.27 | 0.00 | 63.90 | 1.56 | 5.47 | 7.73 | 8.18 | 0.46 | 0.29 | 1.56 | 1.17 | 0.67 | 0.14 |
| LPG130 | 8.06 | 1.34 | 0.00 | 67.69 | 1.66 | 4.34 | 5.99 | 6.38 | 0.49 | 0.30 | 1.65 | 1.24 | 0.71 | 0.14 |
| LPG100 | 8.30 | 1.38 | 0.00 | 69.82 | 4.49 | 5.22 | 1.26 | 2.84 | 1.25 | 1.09 | 2.20 | 1.28 | 0.73 | 0.15 |
| NGL500 | 5.65 | 1.22 | 0.00 | 47.56 | 26.31 | 13.14 | 1.25 | 1.66 | 0.46 | 0.31 | 1.10 | 0.80 | 0.46 | 0.09 |
| NGL430 | 5.96 | 1.25 | 0.00 | 50.20 | 24.33 | 12.13 | 1.18 | 1.57 | 0.47 | 0.32 | 1.16 | 0.86 | 0.49 | 0.10 |
| NGL400 | 6.11 | 1.26 | 0.00 | 51.45 | 23.40 | 11.65 | 1.14 | 1.52 | 0.47 | 0.32 | 1.20 | 0.88 | 0.50 | 0.10 |
| NGL330 | 6.49 | 1.30 | 0.00 | 54.66 | 20.99 | 10.41 | 1.05 | 1.41 | 0.48 | 0.32 | 1.28 | 0.95 | 0.54 | 0.11 |
| NGL300 | 6.68 | 1.31 | 0.00 | 56.19 | 19.84 | 9.82 | 1.01 | 1.36 | 0.49 | 0.33 | 1.32 | 0.98 | 0.56 | 0.11 |
| NGL230 | 7.16 | 1.36 | 0.00 | 60.20 | 16.83 | 8.28 | 0.90 | 1.22 | 0.50 | 0.33 | 1.43 | 1.06 | 0.61 | 0.12 |
| NGL200 | 7.39 | 1.38 | 0.00 | 62.14 | 15.38 | 7.54 | 0.84 | 1.15 | 0.51 | 0.33 | 1.48 | 1.10 | 0.63 | 0.13 |
| NGL100 | 8.31 | 1.47 | 0.00 | 69.80 | 9.64 | 4.60 | 0.63 | 0.88 | 0.54 | 0.35 | 1.68 | 1.26 | 0.72 | 0.15 |
| Test Gas | 9.53 | 1.59 | 0.01 | 80 | 1.96 | 0.66 | 0.34 | 0.52 | 0.58 | 0.36 | 1.95 | 1.47 | 0.84 | 0.17 |
|  | N_2_ | CO_2_ | H_2_S | C_1_ | C_2_ | C_3_ | iC_4_ | nC_4_ | iC_5_ | nC_5_ | C_6_ | C_7_ | C_8_ | C_9_ |

**Table S.3.** Assumptions, limitations, and challenges of experimental and modeling works

| **Assumptions:** |  |  |  |  |
| --- | --- | --- | --- | --- |
| - The association energy and volume, the binary interaction coefficients, and mixing rules have been chosen based on the values suggested by  - The adsorption effect was considered only for asphaltene | | | | |
| **Limitations and challenges:** | | | | |
| Test | It measures: | Is it conducted  in porous media? | Output | Limitations |
| VIT | FCM  (second slop of IFT curve) | No | IFT vs. pressure | a drop of oil is contained within a gas chamber and the overall gas-oil composition is not measurable. |
| Slim tube | MCM  (first slop of IFT curve) | Yes | Recovery factor vs. pressure | its output is not directly comparable with the output of the model. |

**References**

1 Teklu, T. W., Alharthy, N., Kazemi, H., Yin, X. & Graves, R. M. in *SPE Western North American and Rocky Mountain Joint Meeting.* (OnePetro).

2 Teklu, T. W. *et al.* Phase behavior and minimum miscibility pressure in nanopores. *SPE Reservoir Evaluation & Engineering* **17**, 396-403, doi:<https://doi.org/10.2118/168865-PA> (2014).

3 Wang, S., Ma, M. & Chen, S. in *SPE Improved Oil Recovery Conference.* (OnePetro).

4 Zhang, K., Jia, N. & Li, S. Exploring the effects of four important factors on oil–CO 2 interfacial properties and miscibility in nanopores. *RSC advances* **7**, 54164-54177, doi:<https://doi.org/10.1039/C7RA10671H> (2017).

5 Zhang, K., Jia, N., Zeng, F. & Luo, P. A new diminishing interface method for determining the minimum miscibility pressures of light oil–CO2 systems in bulk phase and nanopores. *Energy & Fuels* **31**, 12021-12034, doi:<https://doi.org/10.1021/acs.energyfuels.7b02439> (2017).

6 Sugden, S. CXLII.—A relation between surface tension, density, and chemical composition. *Journal of the Chemical Society, Transactions* **125**, 1177-1189, doi:<https://doi.org/10.1039/CT9242501177> (1924).

7 Zhang, K., Nojabaei, B., Ahmadi, K. & Johns, R. T. in *Unconventional Resources Technology Conference, Houston, Texas, 23-25 July 2018.* 2462-2476 (Society of Exploration Geophysicists, American Association of Petroleum …).

8 Zhang, K., Jia, N., Li, S. & Liu, L. Nanoscale-extended correlation to calculate gas solvent minimum miscibility pressures in tight oil reservoirs. *Journal of Petroleum Science and Engineering* **171**, 1455-1465, doi:<https://doi.org/10.1016/j.petrol.2018.08.057> (2018).

9 Mohammad, R., Zhang, S., Haq, E., Zhao, X. & Lu, S. in *IOP Conference Series: Earth and Environmental Science.* 012030 (IOP Publishing).

10 Song, Y., Song, Z., Guo, J., Feng, D. & Chang, X. Phase behavior and miscibility of CO2–hydrocarbon mixtures in shale nanopores. *Industrial & Engineering Chemistry Research* **60**, 5300-5309, doi:<https://doi.org/10.1021/acs.iecr.1c00717> (2021).

11 Song, Y. *et al.* in *SPE Improved Oil Recovery Conference.* (OnePetro).

12 Sun, H. & Li, H. Minimum miscibility pressure determination in confined nanopores considering pore size distribution of tight/shale formations. *Fuel* **286**, 119450, doi:<https://doi.org/10.1016/j.fuel.2020.119450> (2021).

13 Ahmadi, K. & Johns, R. T. Multiple-mixing-cell method for MMP calculations. *SPE journal* **16**, 733-742 (2011).

14 Abudour, A. M., Mohammad, S. A., Robinson Jr, R. L. & Gasem, K. A. Volume-translated Peng-Robinson equation of state for liquid densities of diverse binary mixtures. *Fluid Phase Equilibria* **349**, 37-55, doi:<https://doi.org/10.1016/j.fluid.2013.04.002> (2013).

15 Zhang, K. & Gu, Y. Two different technical criteria for determining the minimum miscibility pressures (MMPs) from the slim-tube and coreflood tests. *Fuel* **161**, 146-156, doi:<https://doi.org/10.1016/j.fuel.2015.08.039> (2015).

16 Sun, H. & Li, H. A modified cell-to-cell simulation model to determine the minimum miscibility pressure in tight/shale formations. *Oil & Gas Science and Technology–Revue d’IFP Energies nouvelles* **76**, 48, doi:<https://doi.org/10.2516/ogst/2021031> (2021).

17 Metcalfe, R., Fussell, D. & Shelton, J. A multicell equilibrium separation model for the study of multiple contact miscibility in rich-gas drives. *Society of Petroleum Engineers Journal* **13**, 147-155, doi:<https://doi.org/10.2118/3995-PA> (1973).

18 Fazelabdolabadi, B. & Alizadeh-Mojarad, A. On the adsorption and hydrodynamics behavior of H2S and CO2 molecules in organic liquids inside nanoslit pores in vicinity of calcite {101¯ 4} surface. *Journal of Natural Gas Science and Engineering* **28**, 106-120, doi:<https://doi.org/10.1016/j.jngse.2015.11.023> (2016).

19 Fazelabdolabadi, B. & Alizadeh-Mojarad, A. A molecular dynamics investigation into the adsorption behavior inside {001} kaolinite and {1014} calcite nano-scale channels: the case with confined hydrocarbon liquid, acid gases, and water. *Applied Nanoscience* **7**, 155-165, doi:<https://doi.org/10.1007/s13204-017-0563-1> (2017).

20 Jin, B. & Nasrabadi, H. Phase behavior of multi-component hydrocarbon systems in nano-pores using gauge-GCMC molecular simulation. *Fluid Phase Equilibria* **425**, 324-334, doi:<https://doi.org/10.1016/j.fluid.2016.06.018> (2016).

21 Xu, J., Yuan, Y., Xie, Q. & Wei, X. 01124 (EDP Sciences).

22 Peng, F., Wang, R., Guo, Z. & Feng, G. Molecular dynamics simulation to estimate minimum miscibility pressure for oil with pure and impure CO2. *Journal of Physics Communications* **2**, 115028, doi:<http://dx.doi.org/10.1088/2399-6528/aaf090> (2018).

23 Cui, W. *et al.* Estimation of the minimum miscibility pressure for CO2–crude-oil systems by molecular dynamics simulation. *Petroleum Research* **8**, 1-10, doi:<https://doi.org/10.1016/j.ptlrs.2022.08.001> (2023).

24 Doryani, H., Kazemzadeh, Y., Parsaei, R., Malayeri, M. R. & Riazi, M. Impact of asphaltene and normal paraffins on methane-synthetic oil interfacial tension: An experimental study. *Journal of Natural Gas Science and Engineering* **26**, 538-548, doi:<https://doi.org/10.1016/j.jngse.2015.06.048> (2015).

25 Escrochi, M., Mehranbod, N. & Ayatollahi, S. The gas–oil interfacial behavior during gas injection into an asphaltenic oil reservoir. *Journal of Chemical & Engineering Data* **58**, 2513-2526, doi:<https://doi.org/10.1021/je400403y> (2013).

26 Ghorbani, M. *et al.* Experimental Investigation of Asphaltene Content Effect on Crude Oil/CO2 Minimum Miscibility Pressure. *Periodica Polytechnica Chemical Engineering* **64**, 479-490, doi:<https://doi.org/10.3311/PPch.15980> (2020).

27 Hassanpour, S., Malayeri, M. R. & Riazi, M. Utilization of Co3O4 nanoparticles for reducing precipitation of asphaltene during CO2 injection. *Journal of Natural Gas Science and Engineering* **31**, 39-47 (2016).

28 Kazemzadeh, Y., Eshraghi, S. E., Riazi, M. & Zendehboudi, S. How do metal oxide nanoparticles influence on interfacial tension of asphaltic oil-Supercritical CO2 systems? *The Journal of Supercritical Fluids* **135**, 1-7, doi:<https://doi.org/10.1016/j.supflu.2017.12.007> (2018).

29 Kazemzadeh, Y., Parsaei, R. & Riazi, M. Experimental study of asphaltene precipitation prediction during gas injection to oil reservoirs by interfacial tension measurement. *Colloids and Surfaces A: Physicochemical and Engineering Aspects* **466**, 138-146, doi:<https://doi.org/10.1016/j.colsurfa.2014.10.053> (2015).

30 Lu, T., Li, Z., Fan, W., Zhang, X. & Lv, Q. Nanoparticles for inhibition of asphaltenes deposition during CO2 flooding. *Industrial & Engineering Chemistry Research* **55**, 6723-6733, doi:<https://doi.org/10.1021/acs.iecr.5b04893> (2016).

31 Safaei, A. & Riazi, M. Estimating the minimum miscibility pressure (MMP) of methane-live oil using the slim tube test, by modified oil recovery factor (MORF) and break-over pressure (MBOP) criteria. *Petroleum Science and Technology* **41**, 713-730, doi:<https://doi.org/10.1080/10916466.2022.2069817> (2023).
